# Costly teaching contributes to the acquisition of spear hunting skill among BaYaka forager adolescents

**DOI:** 10.1098/rspb.2022.0164

**Published:** 2022-05-11

**Authors:** Sheina Lew-Levy, Daša Bombjaková, Annemieke Milks, Francy Kiabiya Ntamboudila, Michelle Anne Kline, Tanya Broesch

**Affiliations:** ^1^ Department of Human Behavior, Ecology and Culture, Max Planck Institute for Evolutionary Anthropology, Leipzig, Germany; ^2^ Department of Comparative Cultural Psychology, Max Planck Institute for Evolutionary Anthropology, Leipzig, Germany; ^3^ Institute of Social Anthropology, Faculty of Social and Economic Sciences, Comenius University, Bratislava, Slovakia; ^4^ Department of Archaeology, University of Reading, Reading, UK; ^5^ Faculté des Lettres, Arts, et Sciences Humaines, Marien Ngouabi University, Brazzaville, Republic of the Congo; ^6^ Division of Psychology and Centre for Culture and Evolution, Brunel University, Uxbridge, UK; ^7^ Department of Psychology, Simon Fraser University, Burnaby, Canada

**Keywords:** evolution of teaching, hunter–gatherers, spear hunting, adolescence, cumulative culture

## Abstract

Teaching likely evolved in humans to facilitate the faithful transmission of complex tasks. As the oldest evidenced hunting technology, spear hunting requires acquiring several complex physical and cognitive competencies. In this study, we used observational and interview data collected among BaYaka foragers (Republic of the Congo) to test the predictions that costlier teaching types would be observed at a greater frequency than less costly teaching in the domain of spear hunting and that teachers would calibrate their teaching to pupil skill level. To observe naturalistic teaching during spear hunting, we invited teacher–pupil groupings to spear hunt while wearing GoPro cameras. We analysed 68 h of footage totalling 519 teaching episodes. Most observed teaching events were costly. Direct instruction was the most frequently observed teaching type. Older pupils received less teaching and more opportunities to lead the spear hunt than their younger counterparts. Teachers did not appear to adjust their teaching to pupil experience, potentially because age was a more easily accessible heuristic for pupil skill than experience. Our study shows that costly teaching is frequently used to transmit complex tasks and that instruction may play a privileged role in the transmission of spear hunting knowledge.

## 1. Introduction

While other species evidence cultural traditions [1], the human capacity for cumulative cultural evolution is unparalleled [2–5]. Our propensity for high-fidelity transmission may be necessary to the accumulation of cultural knowledge because it increases trait longevity, resulting in more opportunities for modification and diversification [6,7]. Teaching is a type of high-fidelity transmission in which a teacher facilitates learning in a pupil, often at a cost [8–11]. For example, teaching may be energetically costly to perform, may result in the potential loss of resources, or may be time consuming [9]. Considering these costs, theorists have demonstrated that teaching is likely to evolve when complex fitness-enhancing cultural traits are not easily acquired through other social learning mechanisms (e.g. imitation, observation) [8,12–15]. Thus, the pervasiveness of teaching across human societies [8,16], as well as aspects of our social cognition including ostensive cueing [13], joint attention [2], language [17,18] and sensitivity to pupil knowledge and needs [19] may reflect species-specific adaptations for enhancing the acquisition of highly efficient but difficult-to-learn knowledge and skill [12,15].

Different teaching types may vary in cost based on the degree to which the teacher must interrupt their own activities, monitor pupil behaviour and modify their behaviour to facilitate pupil learning [8,20]. Teachers may use less costly forms of teaching (e.g. feedback, teasing) across a range of contexts to facilitate pupil access or attention to relevant stimuli. For complex tasks, which are usually more dangerous or difficult, teachers may promote both access and attention through costlier teaching methods (e.g. instruction, demonstration). While interview studies conducted among Yasawans [21] and BaYaka and Hadza foragers [22] found only weak associations between the complexity of a task and the likelihood that it would be learned via teaching, these studies focused on holistic conventional definitions for teaching used by participants and did not distinguish between teaching types. More costly teaching types, such as verbal instruction, have been shown to improve the transmission fidelity and efficacy of complex manufacturing tasks over and above imitation and emulation in experimental settings [17,23,24]. The present study aims to empirically compare how different teaching types varying in cost contribute to the transmission of spear hunting, a complex task.

The efficiency and longevity of spear hunting raise the possibility that our species' cognitive capacity for especially costly but high-fidelity teaching may have co-evolved alongside this complex task. Archaeological evidence of hand-delivered thrusting and throwing spears dates to the Middle Pleistocene [25] while evidence for hominins throwing and hunting may date even earlier [26,27]. Like other hunting techniques, much of the knowledge necessary for hunting with spears, including tool manufacturing, animal behaviour, environmental cues and hunting strategies [28], must be sequentially acquired for success and are causally opaque in the sense that underlying mechanisms cannot be easily observed or inferred. Across cultures, hunting skill peaks in mid-adulthood [29]. Experimental research further suggests that spear hunting may take longer to learn than other forms of hunting (e.g. bows, atlatls) [30,31]. In sum, the difficulty, causal opacity and long investments in learning associated with spear hunting imply that the benefits of costly teaching, such as verbal instruction, may be relatively large [13], especially considering the high net lifetime production associated with hunting returns [32]. Only Dira & Hewlett [33] have previously investigated how teaching contributes to spear hunting skill acquisition. Based on four focal follows, the authors found that Ethiopian Chabu forager adults frequently taught adolescents during spear hunts, and that verbal instruction was the most common form of teaching observed.

Building on this work, the present study used video and interview data to investigate how teaching contributes to the acquisition of spear hunting knowledge among BaYaka forager adolescents. We focused on adolescents because spear hunting likely requires a long time investment to acquire the requisite motor skills and body size to achieve accuracy and power [31,34,35] and because complex task acquisition appears to be especially important during this life stage [36,37]. Considering the theoretical association between teaching, task complexity and task efficiency [8,12–15], we predicted that in the domain of spear hunting, costlier teaching would be observed at a greater frequency than less costly teaching. Reflecting species-specific adaptations for enhancing teaching efficacy by building upon pupils’ existing competencies [19], we predicted that teachers would be sensitive to pupil skill, as evidenced by older pupils and those with more spear hunting experience receiving less teaching and being given more opportunities to lead the spear hunt.

## 2. Ethnographic background

Data for the present study were collected along the Motaba River in the Likouala Department of the Republic of the Congo. At the study site, BaYaka foragers spend approximately six months of the year in forest camps, and the remaining year in a multi-ethnic village [38]. In both settings, BaYaka participate in day-long and overnight foraging excursions where they fish, hunt with spears and shotguns, set traps and snares, collect honey, wild yams, nuts, mushrooms and greens, and maintain small horticultural gardens [39,40]. While in the village, BaYaka performs agricultural labour for neighbouring Bandongo fisher–farmers in exchange for cultigens and market goods. Approximately 70% of BaYaka diets are from non-domesticated resources, with the remaining 30% coming from locally produced cultigens [41]. Food taboos still practiced at the field site prohibit BaYaka from consuming domesticated animals [42]. Thus, hunting continues to play a central role in BaYaka subsistence.

While BaYaka men often hunt with shotguns owned by neighbouring Bandongo farmers, the meat obtained is given back to the gun owner in exchange for market goods. Spear hunting, which occurs throughout the year, is a key method with which BaYaka hunt for direct consumption. Prey include brush-tailed porcupine, blue duiker, red duiker, red river hog and historically, elephant [39,43,44]. Spear hunting is practised by groups of adolescents and adult men [39]. Kitanishi [39] reports that group sizes for day-long hunting excursions (*esondo*) range from three to 13 participants (mean = 5.8). Among Congo Basin foragers including BaYaka, learning to hunt with spears starts around the age of 3 years, when children participate in target practice games, pretense play, and rat hunting with lightweight wooden spears [43,45–48]. *In situ* learning starts in early adolescence, when boys accompany fathers and other adult men on spear hunts [22,39,49]. At the study site, access to schooling is limited and BaYaka children continue to be active participants in all subsistence activities [50–52]. Our previous research demonstrated that while spear hunting is widely practised by BaYaka, they consider it to be an especially complex task [22]. We found that most spear hunting knowledge transmission reportedly occurred from fathers and other closely related male kin, primarily via teaching [22,46]. The present study builds on this work by investigating the types of teaching that contribute to BaYaka spear hunting knowledge acquisition.

## 3. Methods

Fieldwork for the present study took place in the larger two BaYaka village neighbourhoods in July and August 2019. Ethical approval was obtained from Simon Fraser University (2019s0187). In-country permission was obtained from the Institut de Recherche en Sciences Exactes et Naturelles. We obtained consent from the community, all participants, and the caregivers of unmarried adolescents prior to the start of research. At the closure of the field season, each participant received a machete to thank them for their time.

### (a) Preliminary adolescent interview

Following BaYaka views on maturity, all unmarried male adolescents and young adults (*n =* 24) inhabiting the larger two village neighbourhoods at the time of data collection participated in an interview (see [46] for full details). We assessed previous adolescent spear hunting experiences by asking them to list the number and species of animals they had successfully spear hunted in the past. Adolescents were asked to free-list from whom they would like to learn spear hunting (*Odinga bane ayekodje we botamboli na gongo*?). We did not ask adolescents to restrict their nominations to adults, but all did. Adolescents named between one and nine adults, averaging 3.33 nominations.

### (b) Sample

Adults were invited to teach spear hunting to an adolescent who had nominated them. Where possible, we invited the first adult listed by the adolescent. We moved down the list if the adult listed did not reside in the community at the time of data collection, was not available to participate due to labour demands, was ill, or had already been invited to teach a different adolescent to spear hunt. Ten adolescents were taught by the first person they nominated, with the remainder taught by individuals in positions 2–7 on their list. Five adolescents were excluded from the study because they left the community shortly after the interview (*n* = 1); they only nominated adults who did not reside in the study community at the time of data collection (*n* = 2); they only nominated an adult who was ill (*n* = 1); or were otherwise unavailable to participate (*n* = 1). In total, our sample consisted of 19 adolescent pupils (*m*_age_ = 15.58, s.d. = 2.17, range: 12–20 years), and 17 adult teachers (*m*_age_ = 38.94, s.d. = 12.16, range: 22–59 years), resulting in 18 teacher–pupil groupings (17 dyads, one triad, one teacher taught twice).

### (c) Teaching follows

Over the span of a month, we invited teacher–pupil groupings to go spear hunting the evening prior to the follow, rescheduling if participants were not available. Participants either used their own spears or selected one of two spears rented by the researcher for the study, consistent with local borrowing practices. When participants alerted the researchers that they were ready to depart, we walked them to the head of the forest trail. There, we fitted both teacher and pupil with GoPro cameras mounted on chest harnesses. We taped over start/stop buttons to avoid disruption of footage. We showed participants how to remove the harness and/or cover the camera should they not want something filmed, or should they be uncomfortable. We filmed follows using GoPros instead of recording observations *in situ* because the researchers were not experienced hunters themselves and thus risked affecting participant hunting success; because film has been used to record subtle forms of teaching among neighbouring Aka [53]; and because playback features of film allowed us to more carefully translate verbal teaching which involved specialist language related to hunting. To make clear the purpose of the hunting trip, we instructed teachers to show the pupil how to spear hunt (*Sesa ye botamboli na gongo*) but did not provide any guidance regarding the teaching methods they should employ. Spear hunting trips lasted on average 3.02 h (range: 1.27–5.28 h). Immediately after the teaching follows, teachers and pupils were separated for a short interview during which they were asked to report whether anyone outside the teacher–pupil grouping had participated in the hunting trip, what animals they had encountered, what they had learned/taught, and by what methods.

### (d) Coding

Due to equipment malfunction, no usable video for three teachers and two pupils (including both the teacher and pupil in one dyad) was recorded. We recorded an average of 127.31 min of video per remaining follow participants (s.d. = 19.72, range: 41.91–150.1), totalling 67.90 h across 300 video files.

Participant footage was considered to start after they had received instructions from the researcher and ended once the researcher turned off the camera and/or removed the harness. Using a continuous interval approach, trained student coders watched all videos and identified sections of footage in which the camera malfunctioned (e.g. the screen was black). All remaining footage was considered *usable.* Trained student coders watched all pupil videos to identify footage in which the participant was *leading,* whereby no one can be seen ahead of the walking pupil. They identified sections of footage in which the pupil was not walking, whereby the participant is sitting or standing. All remaining footage was considered *walking.* These variables were coded in Boris [54].

The first two authors, who are conversational in Yaka, coded teaching. We watched the teacher and pupil videos in tandem and recorded teaching types as events (electronic supplementary material, table S1) in Microsoft Excel. Multiple teaching types could be coded simultaneously. We categorized teaching types that involved direct active teaching and opportunity provisioning as *higher cost* because these interrupt ongoing behaviour, incur a cognitive cost associated with attending to pupil progress, and/or involve additional effort on behalf of the teacher [20]. We categorized teaching types that involved evaluative feedback and enhancement as *lower cost* because these teaching types are compatible with teachers' ongoing behaviours and do not involve monitoring pupils’ ongoing progress. We focused on recording teaching events related to spear hunting and other hunting in which spears are used (e.g. during trapping), as well as general skills related to safely traversing the forest (e.g. wayfinding, weather monitoring). Teaching events were nested within teaching episodes, defined as a sequence of teaching events related to the same topic (e.g. trailing a blue duiker).

Inter-rater reliability for approximately 20% of videos was high (ICC(A,1) greater than or equal to 0.87; electronic supplementary material, table S2). Please see the electronic supplementary material for further detail regarding coder training, coding and inter-coder reliability procedures and deviations.

### (e) Analysis

We tested our hypotheses with a series of multilevel regressions. In all models, observations were at the level of the pupil. To account for repeated observations for teachers, all models included a random effect for teacher.

Model 1 (*teaching cost*) was a binomial regression testing the prediction that higher effort teaching would be observed at a greater frequency than lower effort teaching in the domain of spear hunting. The outcome was the total number of higher cost teaching events, with trials as the number of teaching events a pupil received. Model 2 (*teaching frequency*) was a Poisson regression testing the prediction that older pupils and those with more experience would receive less teaching. The outcome was the total number of teaching events. To account for variation in observation time, we included as an offset the log of maximum usable footage, in hours, within a given grouping. Model 3 (*leading*) was a beta regression testing the prediction that older pupils and those with more experience would be given more opportunity to lead the spear hunt. The outcome was the proportion of walking time in which the pupil was leading the hunt, in minutes.

All models included a fixed effect for pupil age in years estimated using the methods outlined in [46,55], and a fixed effect for pupil experience estimated as the total number of prey previously speared by the pupil (log-transformed as log(*x* + 1)). To facilitate estimation, these variables were *z-*score standardized. To adjust for variation in the presence of more than one pupil during the follow, we included a binary fixed effect for group size (0 = dyad, 1 = triad). Because teaching is a cooperative behaviour, teachers are more likely to reap the inclusive fitness benefits of teaching their kin [12,21]. To adjust for this, we included a fixed effect for degree of relatedness, estimated based on genealogical interviews in the package kinship2 v. 1.8.5 [56]. Please see variable descriptions in table 1. Electronic supplementary material, table S3 shows that while pupil age and pupil experience were correlated, variance inflation factors were low (less than or equal to1.69) suggesting that multicollinearity was not a concern. Models were fit in *Rstan* [57] via *brms* v. 2.14.4 [58] in R v. 4.0.3 [59]. Please see the electronic supplementary material for information regarding model fitting, model checks, deviations from planned analyses and additional analyses.

**Table 1.** Descriptions of variables in the models.

variable	description	type	mean	standard deviation
pupil age^a^	in years	integer	15.58	2.17
pupil total prey speared^b^	count of animals previously spear hunted	integer	7.05	15.61
group size	size of the spear hunting group	0 = dyad, 1 = triad	0.16	0.37
degree of relatedness	genetic relationship between the teacher and pupil	continuous	0.25	0.22

^a^*Z-*score standardized.

^b^Transformed as log(*x* + 1) then *z-*score standardized.

## 4. Results

### (a) Qualitative results

Our dataset comprised 519 teaching episodes totalling 1773 teaching events in the domain of spear hunting. There was a mean count of 3.42 teaching events per episode (s.d. = 4.63, maximum = 38). Instruction and demonstration—both categorized as higher cost—were the most often observed teaching types, at 52% and 13% of all teaching events, respectively (electronic supplementary material, table S1). Higher and lower cost teaching often co-occurred within teaching episodes, with opportunity provisioning and evaluative feedback especially correlated (electronic supplementary material, table S4).

Teachers shared personal hunting stories or told myths about the origins of hunting, thus providing information about animal behaviour, describing common mistakes made by hunters, and contextualizing meat-sharing norms. Teachers demonstrated spear throwing, after which pupils practised this skill while receiving feedback; teachers guided pupils through spear hunting ‘drills’ by instructing them to sequentially stalk, throw, crouch and thrust while walking along the trail; and teachers play-acted hunting scenarios (either as the animal or as another hunter) with the pupil. Teachers actively encouraged pupil participation during the spear hunt by calling attention to tracks or sounds, tasking the pupil with rustling leaves or checking traps, demonstrating duiker calls, teasing the pupil for walking noisily, showing trails and offering heartfelt advice about maintaining hope and courage while hunting.

Groups reported seeing track and sign for an average of 4.28 animals (range: 0–14, s.d. = 3.25). Eight groups (44%) reported having missed prey, though these instances were not caught on camera. While no teachers or pupils successfully captured prey during the hunts, all participants reported that learning had occurred. Both teachers and pupils reported that spear use (e.g. throwing, thrusting), moving with the spear (e.g. walking, stalking) and tracking and trailing were the most frequently taught aspects of spear hunting (electronic supplementary material, table S5). Teachers and pupils reported learning other subsistence skills opportunistically encountered during the spear hunt (e.g. collecting honey, collecting liana fruit—see electronic supplementary material for further discussion). Table 2 shows that only teaching which we categorized as ‘higher cost’ was ever reported by BaYaka participants. Most teachers reported teaching by demonstration, whereas most pupils reported learning via assistance. About half of both teachers and pupils reported teaching/learning through instruction.

**Table 2.** Frequency (percent) of participant interview reports of teaching types given/received during the follow.

teaching type	teacher responses	pupil responses	representative example
instruction	11 (61)	9 (53)	‘teacher told me to pay attention to what he was doing because he wants to show me how to throw the spear’
demonstration	13 (72)	8 (47)	‘teacher explained so that I understood well and then showed me [how to throw] and then I also practised trying to throw’
assistance	7 (39)	12 (71)	‘I gave the pupil the spear so he would try [throwing it] himself’
pedagogical question	1 (6)	2 (12)	‘teacher asked questions to make sure I understood, then demonstrated, then had me demonstrate’

Note that one teacher taught during two follows, and is thus represented twice, and that two pupils declined to respond to the interview questions. Note as well that participants often reported more than one teaching type.

### (b) Model 1—teaching cost

**Figure 1.**
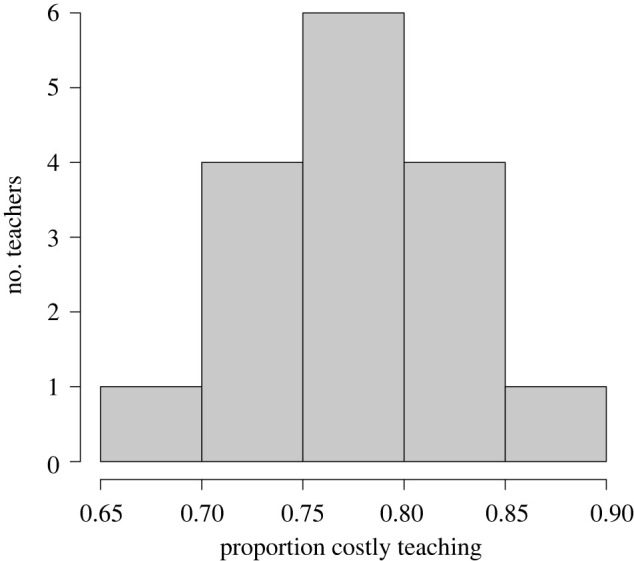
Model 1 random effect estimates for the distribution of teachers' use of costly teaching, as a proportion of all teaching in the domain of spear hunting.

Our prediction that costly teaching in the domain of spear hunting would be observed at a greater frequency than less costly teaching was supported. Based on marginal median estimates, 77.87% (89% credible intervals (CI) = 72.04%, 82.55%) of observed teaching was costly (electronic supplementary material, table S6). Neither pupil age nor total prey speared predicted costly teaching (electronic supplementary material, figure S1). Based on random effect estimates, teachers are predicted to use costly teaching in 68.27–87.19% of all teaching (figure 1). These estimates closely match observed use of costly teaching.

### (c) Model 2—teaching frequency

**Figure 2.**
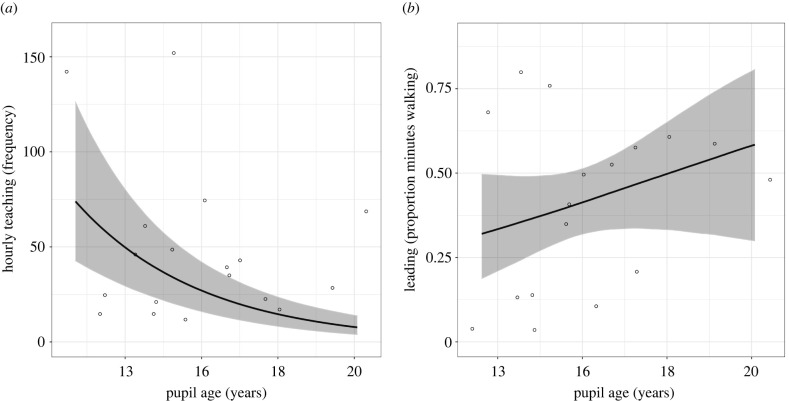
(*a*) Predictions from Model 2 (*teaching frequency*), showing the effect of pupil age in years on the number of hourly teaching events in the domain of spear hunting. (*b*) Predictions from Model 3 (*leading*) showing the effect of pupil age in years on the proportion of walking time during which the pupil led the spear hunt. Experience and degree of relatedness are held at the sample mean, and group size is held at the reference value. Shaded areas represent 89% credible intervals. Scatterplots of observed data are overlaid. Note that while age is positively associated with leading the spear hunt, model results were uncertain, as evidenced by the large credible intervals.

Our prediction that older pupils and those with more spear hunting experience would receive less teaching was partially supported (electronic supplementary material, table S6). Based on marginal median estimates, pupils received 27.39 teaching events an hour. Pupil age was a strong and negative predictor for teaching. Figure 2 shows that with every year increase in age, pupils were 32.81% (89% CI = 22.76%, 44.54%) less likely to receive teaching. Total prey speared by the pupil was not a strong predictor for receiving teaching (electronic supplementary material, figure S2).

### (d) Model 3—leading

Our prediction that older pupils and those with more spear hunting experience would be given more opportunities to lead the hunt was not strongly supported (electronic supplementary material, table S6). Based on marginal median estimates, pupils spent 33.08% (89% CI = 23.31%, 45.80%) of their walking time leading the spear hunt. Though model results were uncertain, and the effect was small, figure 2 shows that every year increase in age was associated with a 3.37% increase in leading the spear hunt (89% CI = −1.93%, 8.88%). Total prey speared by the pupil was not a strong predictor for leading the spear hunt (electronic supplementary material, figure S2).

## 5. Discussion

Using observational and interview data collected among BaYaka foragers, we investigated the role of costly teaching in the transmission of spear hunting knowledge. As predicted, most teaching in the domain of spear hunting was costly. While teachers varied in their use of costly teaching, higher-cost teaching surpassed lower cost teaching in all cases. The prominence of costly teaching was reflected in the post-hunting interviews: both teachers and pupils only reported teaching/learning via higher-cost teaching types. Previous studies have demonstrated that teaching plays a central role in knowledge transmission across a range of contexts and communities [21,36,53,60–65]. Elsewhere, we have argued that foragers generally, and BaYaka specifically, primarily learn through exploration, play, participation and low-cost teaching [51,52,60,66,67]. By distinguishing between different teaching types and their associated costs, the present study extends this body of work by showing that costly teaching—even if rare overall—may be disproportionally used to transmit complex tasks such as spear hunting [8,13].

The high frequency of costly teaching is mostly driven by instruction, which made up about half of our observations for teaching and participant post-hunting interview responses. Other studies investigating teaching in the Congo Basin using similar methods report much lower frequencies for teaching via instruction [53,60,68]. Yet, our findings are comparable with those from Dira & Hewlett [33], who report that verbal instruction made up half of all teaching related to spear hunting among Chabu. While these studies differently operationalized ‘instruction’ (see electronic supplementary material, table S9), all considered instruction to involve the direct and explicit transmission of information. As a teaching type unique to humans [8], instruction appears to play a privileged role in the transmission of spear hunting knowledge above and beyond other subsistence tasks. This may be because instruction improves learning efficiency and enhances skill acquisition [17,24,69,70], even as it constrains exploration [69,71]. Much of the social and ecological knowledge associated with hunting, such as the relationship between animals and their tracks [72], hunter coordination [39] and the effect of sharing norms on animal abundance [42] are causally opaque and thus unlikely to be independently discovered by pupils. By teaching via instruction, teachers may incur higher costs in exchange for ensuring that their pupils rapidly and accurately acquire aspects of spear hunting knowledge that could not be learned by other high-fidelity transmission (e.g. imitation) or even other teaching types [12,14,15].

We also investigated teacher sensitivity to pupil skill. Teaching may be adjusted to pupil skill level such as by ‘providing more and different kinds of instruction when the skill level is low, changing as the learner becomes more skilful, and ceasing when the skill level becomes self-sufficient’ [19]. As predicted, we found that teaching frequency decreased with pupil age, and that older pupils were given more opportunities to lead the spear hunt—though model results for this latter finding were uncertain, and the effect was small. Because increases in age closely track increases in strength, knowledge and experience, age may act as a heuristic for skill [73,74]. Adjusting teaching to pupil skill may improve transmission efficacy by building upon existing competencies [8,19]. As pupils gain skill, they may be given more opportunities to lead without risking the hunt [14]. In sum, sensitivity to pupil skill may maximize pupil learning while minimizing teaching costs [73].

We did not find that teachers were sensitive to pupil experience, measured here as the total prey previously speared by the pupil. There are several potential reasons for this null finding. First, because we did not ask pupils whether adults assisted them during their successful spear hunts and because not all speared animals require the same degree of skill to harvest [75], total prey previously speared may be a noisy measure for skill. Second, pupil responses regarding their experience may have been inaccurate due to recall bias [76]. Finally, it is possible that age is a more accessible heuristic for skill than experience [73]. In an experimental study, Wood *et al*. [77] found that children preferentially copied older individuals, even when they could accurately identify the model's knowledge state. The authors argue that age-based biases are easily adopted heuristics, whereas knowledge-based biases are more cognitively challenging [74,77]. This heuristic hierarchy [78] may be employed by teachers. Assessing pupil experience may be cognitively challenging, requiring the teacher to remember, sometimes over many years, pupil spear hunting successes. Further, while the small-scale nature of this community [79] makes it unlikely that teachers will have no information regarding pupil skill levels, and while peer evaluations have been demonstrated to reliably predict self-reported skill [80], teachers' information regarding pupil experience may nonetheless be incomplete due to prestige avoiding social norms [42], and high rates of mobility [81,82]. As a result, teachers may preferentially weigh their knowledge of pupil age over experience when adjusting their teaching. Controlled experiments are needed to further investigate this possibility.

Our study has several limitations. Despite sampling every adolescent and young adult inhabiting the study community, our sample size was small, reflecting the demographic constraints of BaYaka settlements. We thus sometimes had patchy data, resulting in deviations from our planned analyses (see electronic supplementary material for discussion). It is also unlikely that our statistical analyses could detect small effects with certainty. No groupings successfully captured prey. We thus were not able to observe how teaching occurred in these key moments, though elements involved in catching prey (e.g. stalking, crouching, throwing) were routinely practised while on the trail. We favoured dyadic groupings to track the flow of information from teacher to pupil more easily. While these group sizes were smaller than those usually observed for day-long hunting trips (2–3 in the present study versus 3–13 in Kitanishi [39]), our observations closely reflect how adults report learning to hunt in qualitative narrative interviews (see electronic supplementary material). Because we asked teachers to teach pupils, it is possible that the teaching techniques used reflected what teachers believed researchers wanted to see rather than what would occur in everyday contexts. We find this unlikely because many of the teaching episodes reflected cultural institutions such as *gano* (fables), *mosambo* (public speaking/advice) and *moadjo* (participatory role playing) through which BaYaka teach and enforce social norms, another costly knowledge domain [51,83].

There are many possible extensions of our research. Collecting more precise estimates for experience based on peer and adult rankings, time allocation data spanning several seasons and ecological knowledge tests are needed to improve our understanding regarding how teachers attend to pupil experience when teaching. Research is needed to better understand how spear hunting knowledge transmission occurs in other contexts, such as during evening storytelling [84] and initiation rites [43]. Our cost categorization for teaching types were based on theoretical considerations [20] rather than empirical measures. Future studies could enrich this approach by quantifying time, effort and cognitive cost for specific teaching events on a continuous scale. Finally, future studies should quantitatively investigate how costly teaching and specific teaching types are employed to transmit a range of subsistence and social tasks that vary in required skill, strength and experience.

In summary, our study empirically supports the theory that costly teaching types only observed in humans, such as instruction, may have evolved to transmit complex cumulative knowledge that could not be learned by other means [8,12–15]. Our findings may also suggest that to minimize cost, teachers limit their use of costly teaching to complex tasks and adjust their teaching to pupil skill using easily adopted heuristics. As a complex hunting strategy dating to at least 300 000 BP [25], our findings raise the possibility that the use of spears may have co-evolved with human social cognition including costly teaching, verbal and non-verbal language and teacher–pupil sensitivity.

## Data Availability

The analysis script is provided in the electronic supplementary material [85]. The data are available upon request from the first author.
